# Effect of COGNI-MACHINE computational thinking training on executive functions in children aged 9 to 11: Protocol of a cluster randomized controlled trial

**DOI:** 10.1016/j.mex.2023.102329

**Published:** 2023-08-16

**Authors:** Carolina Robledo Castro, Luz Helena Rodríguez Rodríguez, Luis Fernando Ossa Castillo

**Affiliations:** a Universidad del Tolima, Street 42 #1-02, Ibagué 730006299, Colombia; b Universidad Autónoma de Manizales, Old Railway Station, Manizales 170001, Colombia; c Universidad de Caldas, Street 65 #26-10, Manizales 170002, Colombia; d Universidad Nacional de Colombia Sede Manizales, La Nubia Campus, Manizales 170001, Colombia

**Keywords:** Computational thinking training, Executive functions, Coding, Educational robotics, Cognitive Sciences

## Abstract

We designed a controlled trial protocol that seeks to contribute to cognitive science by studying the effect of thought training on children's executive functions. The study design is a cluster randomized controlled trial, with intra-subject and inter-subject evaluation, with two parallel groups: an experimental group and a TAU control group. With three measures, pre-test, post-test, and follow-up after three months. The participants will be children aged 9 to 11. The allocation will be randomized by groups and not individually. The sample will be a minimum of 44 participants. The primary measures will be neuropsychological tests to assess executive functions. Secondary measures will be a computational thinking test, neuropsychological tests to assess metacognition and attention, and an acceptability scale. The experimental group will participate in the COGNI-MACHINE computational thinking training designed by the first author. The training frequency will be twice a week in 60 min sessions for 12 weeks. The TAU control group will receive computer science classes as usual during the same time as the experimental group. The evaluators taking the measurements will be blinded to the assignment. The investigators in charge of the intervention will be blinded to the results of the evaluations.

## Graphical abstract

**Figure:**
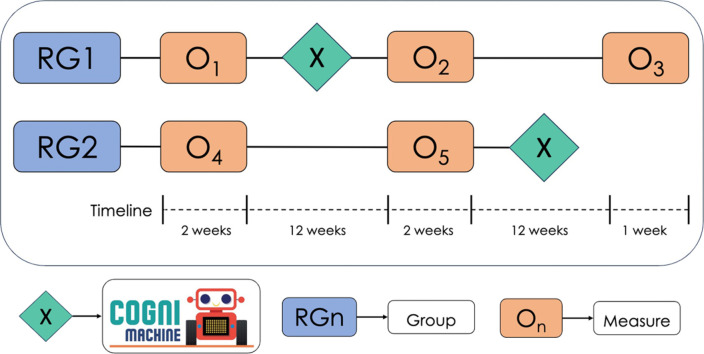

Specifications table

**Table:** 

Subject Area:	• Cognitive sciences
More specific subject area:	• Children's Cognitive Development
Protocol name:	Effect of COGNI-MACHINE computational thinking training on executive functions in children aged 9 to 11: Protocol of a Cluster Randomized Controlled Trial
Reagents/tools:	• Cogni-Machine computational thinking training • Micro:bit based robotics kit • Code.org courses
Experimental design:	The protocol has a Cluster Randomized Controlled Trial design, with intra-subject and inter-subject evaluation, with two parallel groups: experimental and TAU control groups. With three measures, pre-test, post-test, and follow-up after three months. The participants will be four fifth-grade courses, aged 9 to 11. The allocation will be randomized by groups and not individually. The final sample will be a minimum of 44 participants. The primary measures will be neuropsychological tests to assess executive functions (cognitive flexibility, working memory, inhibitory control, and planning). Secondary measures will be a computational thinking test, neuropsychological tests to assess metacognition and attention, a sociodemographic questionnaire, and a satisfaction and adherence scale. The experimental group will participate in the COGNI-MACHINE computational thinking training designed by the first author. The intervention will occur at the educational institution twice a week in 60-minute sessions lasting 12 weeks and 24 sessions. The TAU control group will receive their computer science and technology classes as usual simultaneously and with the same hourly intensity. The evaluators taking the pre-test and post-test measurements will be blinded to the assignment. Moreover, the researchers in charge of the intervention will be blinded to the results of the evaluations.
Trial registration:	The study protocol was registered in International Standard Randomized Controlled Trial Number – ISRCTN, with registration number ISRCTN11380198.
Ethics:	The study protocol was submitted to the Research Ethics Committee of Universidad del Tolima and approved under act No.10 of December 13, 2022.
*Value of the Protocol:	• This protocol is important to verify the effects of COGNI-MACHINE computational thinking training on the development of executive functions of children in the last years of the primary school cycle. • There are some previous studies on the effects of computational thinking on executive functioning in children; however, this is a field of study that requires further development. • The novelty of the COGNI-MACHINE training, compared to other training, is that it seeks to take advantage of the use of unplugged and plugged activities in computational thinking, as well as incorporate educational robotics through project-based learning.

## Introduction

Executive functions (EF) constitute a series of high-level cognitive processes regulating thought, action, and emotion underlie flexible goal-directed responses to new or difficult situations [1], [2], [3]. These cognitive processes depend mainly on the prefrontal areas of the cerebral cortex and their links with other subcortical areas [4] related to the temporal organization of behavior [5]. The prefrontal cortex has a slow and progressive evolution that begins in childhood and reaches its peak in adulthood [[6], [7], [8], [9]]. This means that the development of executive functions is highly dependent on stimulation throughout the development of the subject and that the first years of life are a critical period for acquiring the basic elements that will enhance executive functions throughout life childhood and adolescence [[1], [10], [11], [12]].

The academic community has studied different intervention approaches for executive functions, such as curricular approaches [[13], [14]], programs based on physical activity ([[15], [16]], traditional cognitive training [[17], [18], [19]], cognitive behavioral therapies [[20], [21]], music therapy [22], interventions based on digital technologies [23], [24], [25], [26] , neurofeedback [27], transcranial direct current stimulation [[28], [29]].

In their systematic review, Robledo-Castro et al. [30] compiled studies that evaluated interventions to stimulate executive functions based on computer systems. Among these interventions aimed at children, serious games can be distinguished [[31], [32]], some commercial video games [[33], [34]], programs based on classic tasks [[35], [36]]; interventions that incorporated virtual reality technology [37], among others. The authors also found that research on computational thinking training is another area of study that has shown different effects of teaching coding and programming on children's executive functions [[38], [39], [40], [41], [42], [43], [44], [45], [46], [47], [48], [49], [50], [51]].

Computational thinking refers to a particular way of reasoning to solve problems efficiently following the principles of computational sciences [[52], [53]]. It is a set of cognitive processes involved in a particular approach to interpret, analyze and solve problems [54], which includes: the ability to analyze the problem space, reduce the difficulty of the problem by breaking it down into smaller units, express and represent the solution through algorithms or sequences of instructions, and, finally, correct the execution and verify the scope of the objective [[55], [56], [57], [58]].

One of the tools to develop computational thinking is teaching programming or coding [59]. Knowing the effects of teaching programming and coding on higher cognitive functions has been another line of study that had its first approaches with authors such as Pea & Kurland [60], with variable results. You can also find the meta-analyses [[61], [62], [63]], which jointly compiled 192 studies and evaluated more than a thousand transfer effects of programming learning in different cognitive processes. These meta-analyses concluded that teaching coding and programming at school ages improves computer skills and other higher-order cognitive processes such as critical thinking, mathematical thinking, creative thinking and metacognition, spatial skills, and reasoning.

In recent years, some studies have evaluated the effects of teaching computational thinking on executive functions in childhood. Some of these studies have found small to large effects on executive functions such as planning, inhibitory control, goal-directed behavior, visuospatial working memory, and other cognitive processes such as sequencing, fluid intelligence, problem-solving, verbal and nonverbal reasoning., mathematical skills, reflective thinking, and creative thinking [[38], [39], [43], [45], [46], [49], [50], [64], [65]].

Even though there are some studies on the subject, these are still scarce, and all the authors conclude on the need to strengthen and expand this field of study to provide feedback to existing intervention programs and impact on public and educational policies [46]. Studying the effects of teaching computational thinking on executive functions and other cognitive processes will allow knowing the impact of study plans and programs aimed at teaching computational thinking, recognizing the most appropriate type of activities concerning the ages of the students and their level of development, the real scope of these initiatives and their differential effect in different periods of cognitive development and different executive functions.

### Specific objectives and hypotheses

The following randomized controlled trial (RCT) protocol was designed to evaluate the effect of the COGNI-MACHINE computational thinking training program on the executive functions of fifth-grade children aged 9 to 11 years. This RCT has established the following working hypothesis:

There are significant differences in the evolution of executive functions and other cognitive processes, such as metacognition and attention, in fifth-grade children who receive training in computational thinking compared to those children who receive a conventional computer training program.

## Method

### Protocol design

**Fig. 1:**
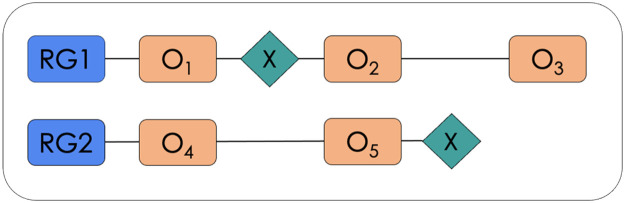
Layout of the experimental design. *Note:* RG1: Experimental group; RG2: Control group; On: Measure; X: Intervention.

A randomized controlled trial will be conducted by groups, with intra-subject and inter-subject evaluation, with two parallel groups, one experimental and the other in active control with treatment as usual. Three measures will be applied: pre-test, post-test, and follow-up at three months. The clinical trial protocol has been designed following the guidelines of the CONSORT Statement [66]. The trial has been registered in the International Standard Randomized Controlled Trial Number - ISRCTN, a primary clinical trial registry recognized by the World Health Organization, with registration number ISRCTN11380198 and can be consulted at: https://www.isrctn.com/ISRCTN11380198. Fig. 1 shows the layout of the experimental design.

For the intra-subject analysis, each group will be evaluated before starting the intervention (T1) and after the intervention (T2). Three months after the end of the intervention, a third follow-up measure (T3) will be applied to the experimental group to recognize the permanence of the effect over time. Once the post-test phase is over, the control group will receive the same training in computational thinking. For the intersubject analysis, the study will include an active control group in treatment as usual (Treatment-As-Usual - TAU), which consists of receiving the usual treatment in parallel to the experimental group and for the same time as the experimental group [[67], [68]].

### Inclusion and exclusion criteria

This study will include children in fifth grade of primary school between 9 and 11 years old. Table 1 contains the criteria for including data from children in the controlled trial.

**Table 1:** Inclusion and exclusion criteria.

Inclusion criteria	Exclusion criteria
1. Children of both sexes.	1. Children with uncorrected sensory impairment.
2. Children who at the time of the beginning the study are between 9 and 11 years old.	2. Children with a psychiatric diagnosis.
3. Children enrolled in fifth grade of primary school in an educational institution of Ibagué, Colombia.	3. Children who exceed the age range at the beginning of the pre-test.
4. Children with typical cognitive development.	4. Children with previous experience in computational thinking, coding or programming training.
5. Children who have participated in 70% of the intervention.	5. Children who have joined the educational institution after the application of the pretest.

### Sample's size calculation

We estimated the size of the groups based on two criteria: (1). A review of previous studies showed a mean group size of 34.8 participants and a median of 26 in similar studies in children [30]. (2) The G-Power projection suggested a group size of 22 children for a total sample of 44 children. Table 2 contains the parameters for the calculation of the sample.

**Table 2:** Parameters for the size of the groups.

Parameter	Value	Description
Effect size	0,25 – 0,5	Data based on reports from previous studies.
α error confidence level	0,05	
Sample power	0,95	
Number of groups	2	1 experimental group and 1 TAU control group
Number of measures	3	Pretest, posttest, follow-up
Sample size	N= 22 per group	N=44 full sample

### Recruitment

We evaluated the possible causes of loss from the sample: (1) subjects who did not meet the inclusion factors for the sample, especially the age of the children; (2) dropout rate for this school grade, which is usually around 15%; consequently, for the recruitment we decided to include 4 fifth grade school groups from the same public educational institution in the city of Ibagué, Colombia. Among the four courses, it is expected to convene approximately 100 children, so that the final sample for the analysis has the minimum projected size.

### Assignment

The intervention will take place in school contexts, and the sessions will be articulated to curricular work in the area of technology; for this reason, it will be necessary to work with naturally constituted school groups. Consequently, the researchers will not individually assign the subjects to the interventions (Experimental and TAU) but will perform cluster randomization. Studies use cluster randomization in circumstances where individual randomization cannot be achieved, so participants are randomized as groups of individuals or clusters [69]. Randomization will be done by an investigator from outside the study who will assign four sealed envelopes (one for each school group) to each intervention.

### Bias control

We used the Cochrane organization's control for bias tool for controlled trials [70] which covers six bias domains. Table 3 shows the actions established to control the appearance of the most common types of bias in non-clinical studies: selection, detection, performance, performance, desertion and reporting [71].

**Table 3:** Cochrane risk of bias check tool.

Bias		Description
Selection bias	Sequence generation	An investigator from outside the study will randomize the school groups to the interventions.
	Allocation concealment	A qualified evaluator outside the research group will be in charge of the evaluation. This evaluator will be blind to the group assignment and the nature of the intervention.
Performance bias	Blinding of participants and staff	The evaluator will be blind to the intervention. Since the control group will be treated as usual, blinding among the participating subjects will not be possible
Detection bias	Blinding of assessors to outcome.	The evaluators will be blind to the type of intervention applied. The investigator in charge of the intervention will be blinded to the results of the evaluation.
Attrition bias	Incomplete outcome data.	Data from participants who complete less than 70% of the training will be excluded from the study. We will perform data imputation; for this, we will evaluate the difference of the results with the data of the excluded subjects and without them.
Reporting bias	Selective reporting of results	We will present the results in accordance with the objectives set out in the trial registration.

### Outcome measures

#### Primary outcome measures

To measure the executive functions of children we have selected neuropsychological tests with recognition and reliability studied. Table 4 contains the selected instruments and the rating parameters.

**Table 4:** Description of primary measures.

Variable	Test	Rating Parameters	Authors
Sequential planning	London Tower	Time since start Execution time Number of moves Number of rule violations	Shallice [72]
Visuospatial planning	Porteus maze, BANFE-2 version.	Number of times it traverses. Number of dead ends Execution time	Flores-Lázaro et al. [73]
Auditory-verbal working memory	WISC-V Digit Scale	Number of right answers in direct order, reverse order and increasing order Digit span in forward order Digit span reverse order Digit span increasing order	Wechsler [74]
Visual spatial working memory	Corsi cubes, version WMS-III	Maximum number of elements Number of correct trials	Corsi [75] Wechsler [76]
Visual working memory	WISC-V Span of drawings Scale	Span of drawings Span of stimuli	Wechsler [74]
Cognitive flexibility	M-WCST Wisconsin cards abridged version	Number of right answers Number of maintenance errors Number of perseverations Number of deferred perseverations Execution time Completed Categories	Arango-Lasprilla et al. [77] Schretlen [78]
Inhibitory control	Classic stroop test	Number of stimuli words, letters and words/letters	Golden [79]

#### Secondary outcome measures

The study will also include secondary variables such as level of satisfaction with training, computational thinking, metacognition, and attention. Table 5 presents the instruments selected to measure these variables.

**Table 5:** Description of secondary measures.

Variable	Test	Rating Parameters	Authors
Computational thinking	CTt scale	Number of right answers	Román-González et al. [[46], [80], [81]]
Metacognition	Metacognition test	Items on a Likert scale. The test is divided into six factors: Knowledge, control and supervision, planning, experiences, evaluation and strategies	Jaramillo-Mora & Osses-Bustingorry [82]
Metamemory	Metamemory scale BANFE-2 version	Number of total errors Number of negative errors Number of positive errors Perseverations Intrusions	Flores-Lazaro et al. [73]
Attention	Trail Making Test TMT D2 Test	Execution time number of errors Number of right answers Number of correct answers minus errors	Portellano y Martínez [83] Brickenkamp, et al. [84]
Satisfaction, acceptability, adherence and feasibility	Satisfaction and adherence scale	Likert scale with 4 response options	Designed by the authors based on Attkisson and Zwick [85]

#### Covariate measures

Sociodemographic and participant-specific factors from both groups will be measured and treated as covariates to describe the population and assess the effectiveness of the intervention. The socioeconomic level (SES) will be estimated with the sociodemographic stratum registry of the National Planning Department (DANE) [86], and other socioeconomic variables, such as parental education, access to technological means, among others, will be evaluated with the Sociodemographic Characterization Questionnaire [87].

### Interventions

#### Experimental group

The experimental group will participate in a training in computational thinking called COGNI-MACHINE, designed by the first author. The intervention comprises 24 sessions; each session will be structured as a workshop and will take place in the computer room of the participating educational institution. The intervention will last 12 weeks, with two sessions per week of 60 min each.

The training workshops will be based on the principles of problem-based learning, and the activities will be designed following the principles of low floor / high ceiling. For the design of the computational thinking intervention, the following sources were taken into account: (1) The recommendations emerging from the results of the previously conducted pilot test [[30], [45]]; (2) A broad review of activities, projects, and courses available to teach computational thinking in school contexts; (3) A systematic review of background and similar studies.

**Fig. 2:**
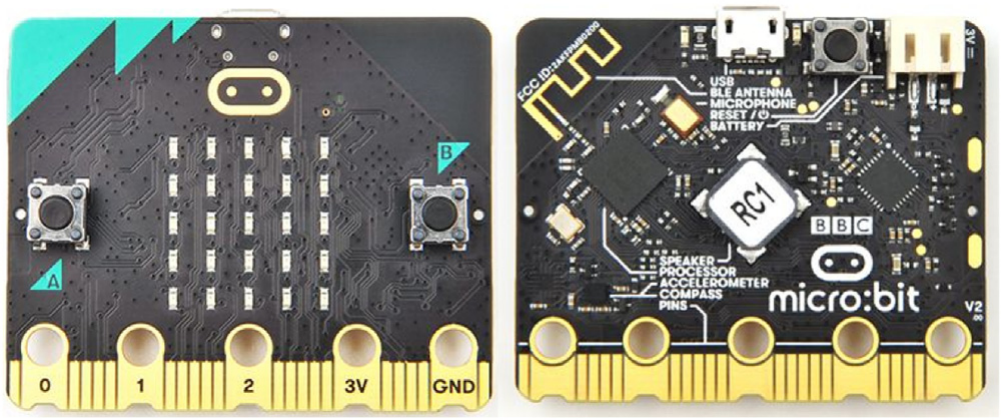
micro:bit board V2.

To select the plugged and unplugged activities that would be part of the training, we reviewed: (1) Existing open-access training and existing repositories of unplugged activities; (2) The integrated development environments available to learn to code in blocks; 3) The educational robotics devices available in the market for Colombia. From these reviews, unplugged activities were selected from different sources such as CS Unplugged, code.org, programemos.com, and Mintic's “Coding for kids” [88]. Lessons from courses B, C, and D of the code.org integrated development platform and the Makecode integrated development platform were selected for the block programming activities. For the plugged projects, he selected the micro:bit V2 microprocessor (see Fig. 2), which is a small programmable board designed by the BBC which has several integrated sensors, Bluetooth connection, radio frequency, push buttons, and LED light outputs.

**Fig. 3:**
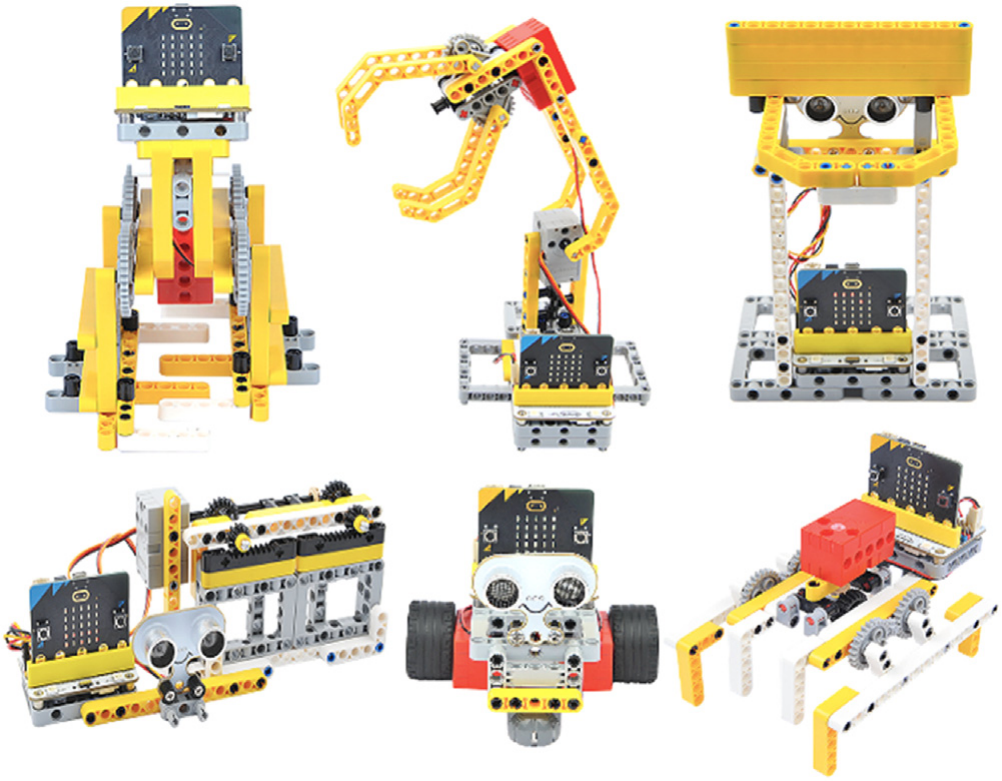
Example of educational robotics projects in micro:bit with the Electricfreaks robotics kit.

For the plugged educational robotics activities, an Electricfreaks micro:bit-based robotics kit was selected (Fig. 3), which has a Wukong expansion board, motors, servomotors, additional external sensors (lone-following, ultrasound, toilet level), buildable tiles, among other supplies to develop different types of projects with the micro:bit microprocessor.

Table 6 summarizes the intervention plan, discriminating the intervention phase, the sessions of each phase, the type of activities conducted, and the computational thinking concepts that will be addressed in said sessions based on the proposed framework by Brennan & Resnick [89].

**Table 6:** Summary of intervention sessions.

Sessions	Intervention phase	Activities	CT concepts							
			Sequences	Loops	Parallelism	Events	Conditionals	Operationals	Variables	Functions
1 to 2	Introduction to computational thinking	- Unplugged activities	x							
3 to 8	Introduction to block language programming and practice of computational thinking skills	- Programming animations and games with courses B, C and D from code.org. - Unplugged activities	x	x		x	x			
9 to l6	Solving problems based on computational thinking	- Development of micro:bit projects programmed in makecode. - Plugged projects	x	x	x	x	x	x	x	x
17 to 24	Robótica educativa	- Educational robotics projects with micro:bit	x	x	x	x	x	x	x	x

#### Control group

The control group at TAU will receive the traditional Technology and Computer Science classes 2 hours a week for 12 weeks. In these classes, students will learn to use Microsoft Office applications. After the 12 weeks of intervention, a crossover design will be conducted so that the control group participates in the same intervention as the experimental group and the experimental group participates in the 12 weeks of traditional technology classes.

### Statistical analysis plan

A hypothesis test will be conducted through Mixed Repeated Measures Analysis of Variance (ANOVA MR) with Bonferroni corrections to look at the differences between groups, in which the pre-test, post-test, and follow-up measures will be the factor of repeated measures. (Within-subject) and the difference between the experimental and control groups as the subject factor. The effect size will be measured with partial eta squared. The assumptions of this type of analysis assume a continuous metric-dependent variable with approximately normal distribution and with homoscedasticity in each of the groups.

A multivariate linear regression analysis will also be performed to assess the correlations between cognitive measures and computational thinking. And logistic regressions to analyze binary variables to evaluate the association between the covariates and the results.

### Ethical considerations

The study complies with all the ethical requirements described in the Helsinki Declaration and Resolution 8430, which establishes the norms for health research, according to which this study would be classified as without risk [90]. Responding to these parameters, the researchers built informed consent for the parents and guardians and informed assent for the children. These documents will be signed by all the participants who agree to be part of the study. Additionally, a protocol was established with risk minimization actions. This randomized controlled trial has the endorsement of the Bioethics Committee of the University of Tolima registered in Minutes No. 10 of December 13, 2022.

## Discussion

Proposing a controlled trial protocol to verify the effects of COGNI-MACHINE computational thinking training on executive functions and other cognitive processes of children in the last years of primary school is relevant for the academic community since it will allow knowing the impact of study plans and programs aimed at teaching computational thinking, recognizing the type of activities that have more acceptability, adherence, and effectiveness in children of this age range as well as recognizing the differential impact that the teaching of computational thinking has on different executive cognitive processes.

One of the main strengths of this study will be the blinding of the evaluators to the allocation and the blinding of the investigators in charge of the intervention to evaluate the subjects. On the other hand, the novelty of the COGNI-MACHINE training, compared to other training, is that it seeks to take advantage of different types of activities to promote computational thinking, not only coding activities. This program includes, on the one hand, disconnected activities to introduce students to the development of more complex and abstract activities. On the other hand, it integrates different types of connected activities, starting with animation activities and game programming in the language of blocks, to later develop robotics projects applied to problem-solving.

One of the study's limitations is that, since the application context is school and integrated into the technology curricular program, individual randomization of the participants will not be possible. Therefore, the randomization will be conducted by groups. Additionally, given the nature of the study, participants will not be blinded to the intervention.

## CRediT authorship contribution statement

**Carolina Robledo Castro:** Conceptualization, Methodology, Validation, Formal analysis, Investigation, Project administration, Resources, Writing – original draft. **Luz Helena Rodríguez Rodríguez:** Conceptualization, Formal analysis, Investigation, Project administration, Resources. **Luis Fernando Ossa Castillo:** Methodology, Formal analysis, Supervision, Writing – review & editing.

## Data Availability

Data will be made available on request.
